# Natural Variation in Seed Very Long Chain Fatty Acid Content Is Controlled by a New Isoform of KCS18 in *Arabidopsis thaliana*

**DOI:** 10.1371/journal.pone.0049261

**Published:** 2012-11-08

**Authors:** Sophie Jasinski, Alain Lécureuil, Martine Miquel, Olivier Loudet, Sylvain Raffaele, Marine Froissard, Philippe Guerche

**Affiliations:** 1 INRA, UMR1318, Institut Jean-Pierre Bourgin, RD10, Versailles, France; 2 AgroParisTech, Institut Jean-Pierre Bourgin, RD10, Versailles, France; 3 The Sainsbury Laboratory, Norwich Research Park, Norwich, United Kingdom; Max Delbrueck Center for Molecular Medicine, Germany

## Abstract

Oil from oleaginous seeds is mainly composed of triacylglycerols. Very long chain fatty acids (VLCFAs) are major constituents of triacylglycerols in many seed oils and represent valuable feedstock for industrial purposes. To identify genetic factors governing natural variability in VLCFA biosynthesis, a quantitative trait loci (QTL) analysis using a recombinant inbred line population derived from a cross between accessions Bay-0 and Shahdara was performed in *Arabidopsis thaliana*. Two fatty acid chain length ratio (CLR) QTL were identified, with one major locus, *CLR.2*, accounting for 77% of the observed phenotypic variation. A fine mapping and candidate gene approach showed that a key enzyme of the fatty acid elongation pathway, the β-ketoacyl-CoA synthase 18 (KCS18), was responsible for the *CLR.2* QTL detected between Bay-0 and Shahdara. Association genetics and heterologous expression in yeast cells identified a single point mutation associated with an alteration of KCS18 activity, uncovering the molecular bases for the modulation of VLCFA content in these two natural populations of Arabidopsis. Identification of this *kcs18* mutant with altered activity opens new perspectives for the modulation of oil composition in crop plants.

## Introduction

Seed storage compounds are of vital importance both for the human diet and industrial uses. Protein-rich seeds are mainly used for food, whereas oil from oleaginous crops has a larger spectrum of uses. Indeed, seed lipids, which are composed of triacylglycerols (TAG) in most plant species, are structurally similar to long chain hydrocarbons derived from petroleum, and thus constitute excellent renewable resources for the production of oleochemicals [1], [2]. Consequently, they have the potential to fulfill market needs in a wide range of industrial sectors, including food, health, cosmetics and the chemical industry. Due to the economic importance of vegetable oils and their expanded use as a renewable feedstock, there is considerable interest in increasing total seed oil yield and in optimizing the fatty acid composition of industrially important oils in high-yield crops. Depending on the use of the oil, different fatty acid compositions are needed and one major objective for plant biotechnologies is to take advantage of the extraordinary diversity of plant fatty acids to produce oils with the desired fatty acid composition. For that purpose, a detailed understanding of fatty acid biosynthesic and TAG assembling pathways is crucial.

In recent years, much progress has been made in unraveling the enzymatic pathways of plant fatty acid and oil synthesis and in identifying the genes that encode the key enzymes of these pathways in several oilseed species [3], [4]. As a result, transgenic lines were produced in an attempt to manipulate the content or quality of seed oils; oil quality was modified either by alteration of the relative proportions of the main common fatty acids or by production of unusual fatty acids by introduction of complex multistep biosynthetic pathways from another species [5], [6], [7], [8], [9]. If some of these approaches were successful, most of the attempts reported so far resulted in a relatively small increase in oil content or in the accumulation of low concentrations of the desired fatty acids, making these crops unsuitable for cost-effective industrial use on a large scale [7], [8]. A complementary approach to reverse genetics to manipulate the content and/or quality of seed oil is to analyze natural variation. Indeed, in the last decade, natural variation studies greatly contributed to our understanding of plant development and physiology including for example seed dormancy and germination, flowering time, plant architecture and morphology or primary metabolism [10]. Historically, breeders have exploited natural variation in oil composition in crops like rapeseed, which is the most widely grown crop for the production of vegetable oils in Europe (http://www.prolea.com). In rapeseed, natural variation is exploited through the use of two types of cultivars: low erucic acid rapeseed (LEAR, containing almost 0% erucic acid) used for human consumption, and high erucic acid rapeseed (HEAR, containing approximately 50% erucic acid) used for industrial purposes. Very long chain fatty acid (VLCFA, i.e., fatty acid containing more than 18 carbon atoms), like erucic acid, are synthesized from C18 carbon chains by sequential elongation reactions performed by a fatty acid elongation complex bound to the endoplasmic reticulum membrane. Each elongation reaction consists of four steps: (i) the condensation of two carbons from malonyl-CoA to an acyl-CoA catalyzed by a fatty acid elongase (β-ketoacyl-CoA synthase, KCS), (ii) a reduction reaction catalyzed by a β-ketoacyl-CoA reductase (KCR), (iii) a dehydration reaction performed by a β-hydroxyacyl-CoA dehydratase (HCD), and (iv) a reduction reaction performed by an enoyl-CoA reductase (ECR) [11], [12]. VLCFAs represent an important quantitative component of TAG in seeds of the brassicaceae family. Thus, a major challenge is to understand the molecular mechanisms that regulate the biosynthesis of these VLCFAs to modulate their proportion in the oil of industrial crops.

The relative proportion of each fatty acid in seed oil, like many traits of agronomic importance, has a complex genetic basis and is quantitative in nature. The identification of quantitative trait loci (QTL) is a first step towards dissecting the molecular basis of such complex traits. Indeed, quantitative genetics has been used to search for genetic factors controlling oil quality in a variety of plants including rapeseed [13], [14], [15], [16], [17], soybean [18], oil palm [19], maize [20], [21], [22], rice [23], wheat [22], Arabidopsis [24], [25] and even more recently Jatropha [26]. However, except for one QTL affecting seed oil and oleic acid content in maize [21] and three QTL affecting oil composition in canola [13], most of these studies did not go beyond the mapping stage. There are a variety of different reasons that could result in failure to clone a QTL, including difficulties to accurately phenotype for the character analyzed, to obtain enough recombinant individuals to shorten the QTL area, to develop efficient markers to genotype individuals, or to identify relevant candidate genes. Because QTL cloning is easier in model species for which substantial genetic resources exist, we implemented a QTL approach to study lipid metabolism in Arabidopsis seed. Indeed, Arabidopsis has been developed as a model system for the genetic analysis of lipid biosynthesis [27], [28]. Investigating natural variation can reveal the allelic richness in nature, which results in a variety of protein structures and/or activities adapted for plant life. Studying these natural variants has the potential to improve our understanding of their mode of action and/or their regulation. In addition, lipid metabolism is very similar between Arabidopsis and Brassica species and the close relationship between them allows the use of comparative genetics to predict orthologous genes and alleles within the Brassica genome [29]. This will enable the transfer of discoveries from Arabidopsis into crop breeding programs.

In this study, a search for genetic factors governing natural variation in VLCFA content in Arabidopsis seed was carried out. The fatty acid chain length ratio (CLR: the ratio between the total amount of C20 to 24 fatty acids and the total amount of C16 to 18 fatty acids) is a robust indicator of VLCFA proportion [30]. As a quantitative trait, variation in the CLR was studied through QTL analysis in Arabidopsis recombinant inbred lines (RILs) derived from the Bay-0 and Shahdara accessions, which display a contrasted CLR phenotype (this study and [30]). Here, we describe the identification of a QTL on chromosome 4, *CLR.2*, controlling 77% of the CLR trait in the RIL set. The *CLR.2* QTL was fine mapped and the causal factor was shown to be a mutation in the *KCS18* gene, also known as *FAE1*, encoding a β-ketoacyl-CoA synthase protein belonging to the fatty acid elongase complex. Association genetics identified the polymorphism that underlies CLR variation among Arabidopsis accessions, revealing a new *KCS18* allele with altered activity.

## Results

### The *CLR.2* QTL Accounted for 77% of Fatty Acid Chain Length Ratio Variation in the Bay-0 x Shahdara RIL Population

A quantitative genetics approach was used to investigate natural genetic variation for fatty acid chain length ratio (CLR: the ratio between the total amount of C20 to 24 fatty acids and the total amount of C16 to 18 fatty acids) as a complex trait. To this end, we used the previously described Bay-0 x Shahdara recombinant inbred line (RIL) population [31], the parental accessions of which displayed a contrasted fatty acid composition resulting in a contrasted CLR phenotype (Fig. S1 and [30]). A subset of 164 RILs, optimized for QTL mapping [32] was phenotyped for CLR. These RILs exhibited a wide range of CLR values (Fig. S2), highlighting the potential of this subset to study CLR variation. The RIL set had been genotyped previously for a set of 69 physically anchored microsatellite markers (www.inra.fr/vast/). QTL detection using standard procedures in QTLCartographer (Materials and Methods) was carried out combining the genotypic data available for the 164 RILs with our phenotypic data. Two QTL with LOD scores greater than 2.5 were mapped and named *CLR.1* and *CLR.2* (Table 1). In total, these two QTL explained 85% of the total observed phenotypic variance. The *CLR.2* QTL contributed the most to CLR variation (77% of the total phenotypic variance) and was very highly significant (LOD>56). Thus, fine mapping of *CLR.2* was carried out to determine the cause of the CLR difference observed between Bay-0 and Shahdara.

**Table 1:** QTL detection for CLR in the Bay-0 x Shahdara RIL population.

	Chromosome	Marker	Position (cM)	LOD Score	Additive effect	% of variance
*CLR.1*	3	MSAT3.99	4.8	6.9	0.021	8
*CLR.2*	4	MSAT4.9	58.9	56.2	0.067	77

CLR, fatty acid chain length ratio. The indicated QTL position in centiMorgans (cM) corresponds to the LOD score peak. The additive effect represents the mean effect on CLR of the replacement of both Shahdara alleles by Bay-0 alleles at the QTL.

### Development of a Heterogeneous Inbred Family for Fine Mapping of *CLR.2* QTL

**Figure 1:**
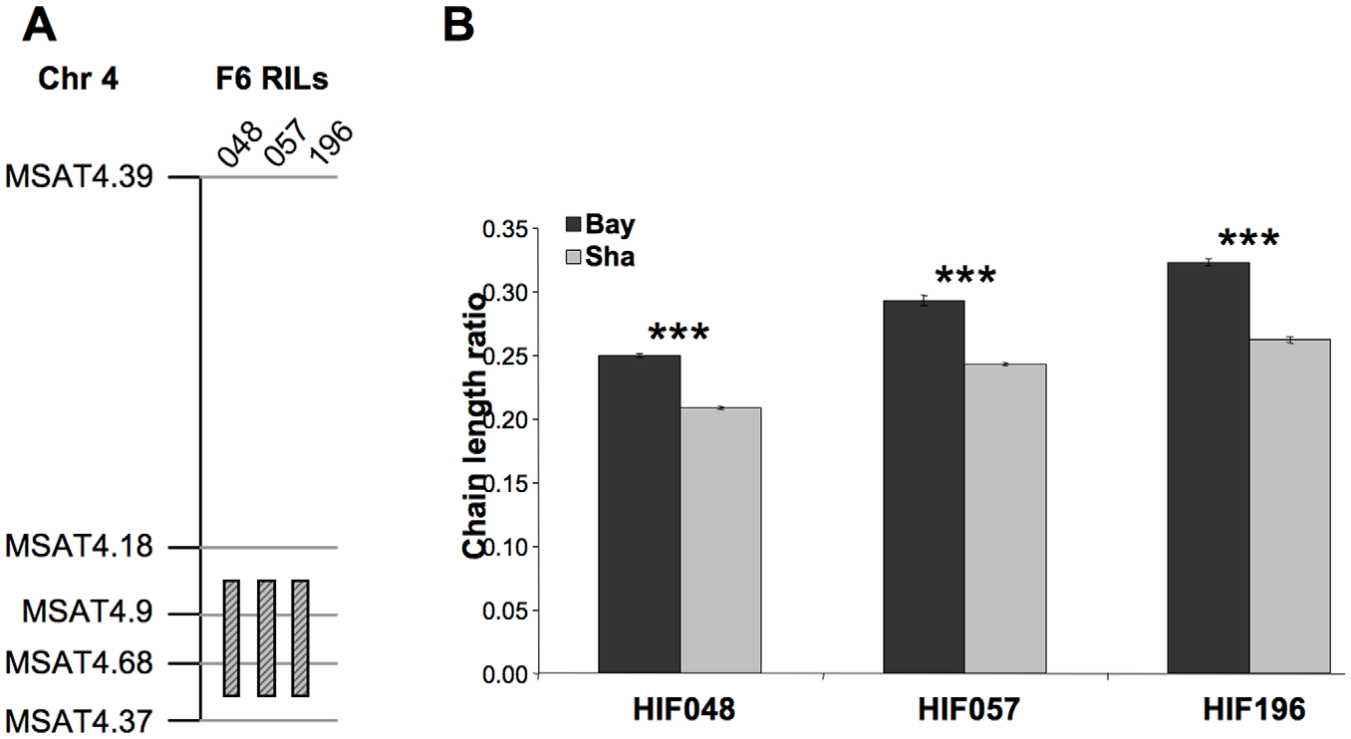
Confirmation of the *CLR.2* QTL with heterogeneous inbred families (HIFs). A, F6 recombinant inbred lines (RILs) from the Bay-0 x Shahdara cross showing residual heterozygosity in the region of the *CLR.2* QTL. Numbers on the top designate the RIL, with the hatched black and grey bar beneath indicating the region still segregating. Recombination breakpoints delimiting heterozygous regions are arbitrarily depicted in the middle of the marker interval. The vertical black line represents chromosome 4 with markers indicated on the left. Marker positions and identity can be found at www.inra.fr/vast/in the MSAT database. B, Comparison of CLR for HIFs fixed for the Bay (black) or the Sha (grey) allele at the segregating region. CLR was determined from 20 seeds per plants. Bars represent SE values (n = 8, 4 repetitions were done on 2 plants). Significance in t-test, ***p<10^−8^.

The phenotypic effect linked to *CLR.2* was confirmed using specific near-isogenic lines differing for a small genomic region spanning a few Mb around the QTL. Near-isogenic lines for this QTL were obtained by producing heterogeneous inbred families (HIFs), which are easily generated taking advantage of the residual heterozygosity still segregating in F6 RILs [33], [34]. Three candidate RILs (048, 057 and 196), segregating only around *CLR.2* but fixed as homozygous for all the tested markers in the rest of chromosome 4, were used to generate HIFs (Fig. 1A). All three HIFs developed from these RILs (Materials and Methods) showed a significant difference in the level of CLR between plants bearing the allele from Bay-0 (“Bay”) and plants bearing the allele from Shahdara (“Sha”) (Fig. 1B). Furthermore, the three HIFs exhibited the expected higher level of CLR associated with the Bay allele with respect to the Sha allele. The extent of the phenotypic change was also in agreement with the QTL analysis.

### *KCS17* and *KCS18* were Candidate Genes underlying the *CLR.2* QTL

**Figure 2:**
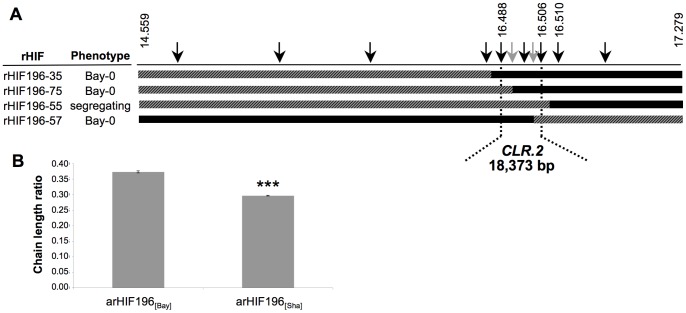
Fine mapping of *CLR.2*. A, The genotype of the most informative recombinants (rHIFs) used to delineate the final 18,373-bp candidate interval are represented with horizontal bars (black for Bay allele, grey for Sha allele, hatched black/grey for heterozygous). Vertical black arrows above represent markers used for genotyping between 14.559 Mb and 17.279 Mb on chromosome 4. Numbers indicate physical position in Mb (TAIR8). Grey arrows mark the most informative recombination breakpoints. B, Chain length ratio of arHIF196_[Bay]_ and arHIF196_[Sha]_ (Materials and Methods) was compared. CLR was determined on 100 seeds from 4 independent plants for each arHIF. Bars represent SE values (n = 4). Significance in t-test, ***p<10^−6^.

HIF196 was used further for fine mapping of *CLR.2* since it was heterozygous only around *CLR.2* and homozygous for the rest of the genome for all the tested markers, whereas RIL048 and RIL057 displayed a second minor heterozygous region on chromosome 5 and 1 respectively. Using additional genetic markers, the heterozygous region of HIF196 was mapped to a 2.7 Mb interval. Screening of 384 F9 progeny plants from a heterozygous F8 RIL196 individual (heterozygous over the 2.7 Mb region) resulted in the isolation of 64 recombination events in this interval. Phenotyping of 13 recombinants (rHIF, see Materials and Methods) reduced the region of interest to a 18 kb interval between markers at positions 16.488 Mb and 16.506 Mb on chromosome 4 (Fig. 2A). Based on some of the most informative recombinants (rHIF196-55 and rHIF196-75), an “advanced rHIF cross” (arHIF; see Materials and Methods and [35]) was designed to obtain the arHIF196 line, which uniquely segregated for a 22 kb region between markers at positions 16.488 Mb and 16.510 Mb, and thus containing the final 18 kb candidate interval. Just like the original HIF, the progeny of this line segregated for CLR with the genotype (arHIF196_[Bay]_ vs. arHIF196_[Sha]_), confirming the presence of *CLR.2* within this interval (Fig. 2B). Seven predicted genes (*At4g34500*, *At4g34510*, *At4g34520*, *At4g34530*, *At4g34540*, *At4g34550* and *At4g34555*) are present in the 18 kb interval (Table S1). A search for predicted or known functions of the encoded proteins revealed that four of these genes were not likely involved in seed lipid metabolism, one had no predicted function and two genes (*At4g34510* and *At4g34520*, further named *KCS17* and *KCS18*) represented good candidates for *CLR.2* since they encode for β-ketoacyl-CoA synthases, involved in the elongation of VLCFA.

### *kcs18* Insertion Mutants Showed Strong CLR Reduction

**Figure 3:**
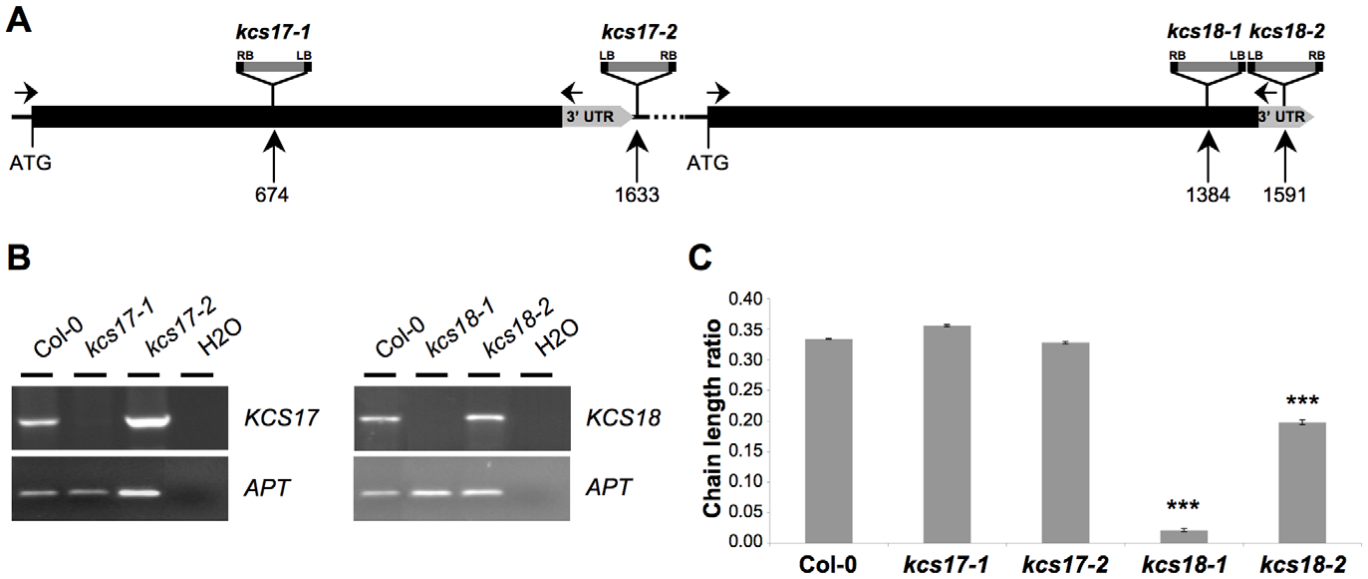
*kcs18* insertion mutants show CLR reduction. A, Structure of the *KCS17* and *KCS18* genes with positions of the T-DNA insertions in *kcs17-1, kcs17-2*, *kcs18-1* and *kcs18-2* mutants indicated. Black boxes represent exons (*KCS17* and *KCS18* are intronless) and grey boxes represent 3′ UTR regions. Numbers indicate distance in basepairs from the ATG. LB, left border of T-DNA; RB, right border of T-DNA. B, RT-PCR analysis of *KCS17* and *KCS18* expression in 10-day-old seeds of wild type (Col-0), *kcs17-1, kcs17-2*, *kcs18-1* and *kcs18-2* plants. *APT* (*ADENINE PHOSPHORIBOSYLTRANSFERASE*) was used as a constitutively expressed gene control. Primers used for RT-PCR are indicated by arrows in A. For each genotype, 15 siliques (5 siliques harvested from 3 different plants) have been dissected. C, CLR from wild type (Col-0) and the indicated *kcs* mutants. *kcs18* mutants display a lower CLR compared to Col-0 whereas *kcs17* mutants display a similar CLR to Col-0. CLR was measured in triplicate on 100 seeds from a pool of 4 independent plants for each genotype. Bars represent SE values (n = 3). Significance in t-test, ***p<10^−5^.

To investigate the possible role of these two genes in CLR variation between Bay-0 and Shahdara, we analyzed T-DNA insertion mutants in *KCS17* (*kcs17-1* and *kcs17-2*) and *KCS18* (*kcs18-1* and *kcs18-2*) available in the Col-0 genetic background (Fig. 3A). Molecular characterization of the mutants revealed that *kcs17-1* contained an insertion 674 nucleotides downstream of the start codon of the *KCS17* gene and *kcs17-2* carried a T-DNA insertion in the intergenic region between *KCS17* and *KCS18*. *kcs18-1* contained an insertion 1384 nucleotides downstream of the start codon of the *KCS18* gene and *kcs18-2* carried a T-DNA insertion in the 3′UTR of *KCS18* gene. RT-PCR analysis of *KCS17* and *KCS18* expression in the respective mutants revealed that full-length *kcs17* mRNA was undetectable in *kcs17-1* plants whereas it was detected in *kcs17-2* plants. Similarly, full-length *kcs18* mRNA was not detected in *kcs18-1* plants whereas it was detected in *kcs18-2* plants (Fig. 3B). Phenotypic analysis for CLR revealed that both *kcs17* homozygous mutants displayed a wild type CLR (Fig. 3C), suggesting that KCS17 is not involved in the CLR phenotype. By contrast, both *kcs18* homozygous mutants had a strongly reduced CLR compared to wild type. *kcs18-1* displayed a much stronger CLR reduction than *kcs18-2* (Fig. 3C), which correlated with the reduction in the level of *KCS18* transcript, suggesting that *CLR.2* is likely due to variation at *KCS18*. Noteworthy, *kcs18-1* is probably a null mutant displaying nearly no elongase activity at all (CLR = 0.02), whereas the Col-0 *kcs18-2* mutant and the Shahdara allele would be considered a hypomorphic (CLR = 0.20 for *kcs18-2* and CLR = 0.29 for Shahdara), still able to elongate fatty acids.

### *CLR.2* Alleles Diversely Complemented the CLR Phenotype of a Null *kcs18* Mutant

**Figure 4:**
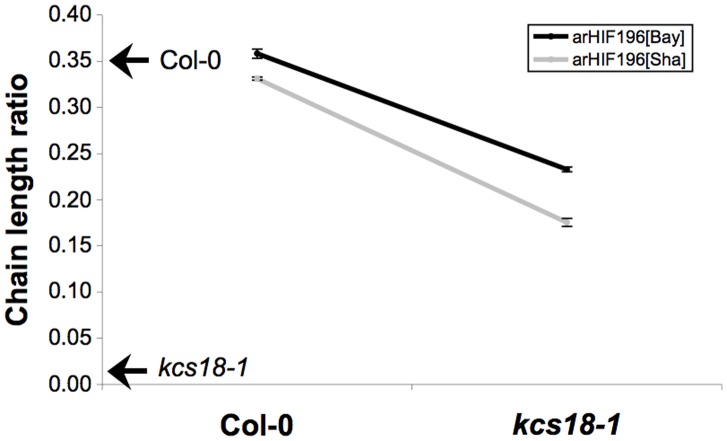
Bay and Sha alleles at *CLR.2* diversely complement *kcs18-1* mutant. CLR was determined for each of the four allelic combinations obtained in F1 seeds, generated by crossing arHIF196_[Bay]_ or arHIF196_[Sha]_ with a *kcs18-1* mutant or its wild type background (Col-0). For each combination, 10 independent crosses have been done and CLR was determined from 100 seeds of each cross. Each data point represents the mean +/− SE (n = 10). CLR values for Col-0 and *kcs18-1* are indicated by an arrow along the y axis. The QTL genotype x gene allele interaction term is very highly significant (p<0.1%).

To further confirm that *KCS18* is *CLR.2*, we performed a quantitative complementation test [36] by crossing both arHIF196 (fixed either Bay or Sha) to the *kcs18-1* mutant or its wild type counterpart (i.e. Col-0) to generate F1 seeds carrying *Bay*/*kcs18-1*, *Sha*/*kcs18-1*, *Bay*/*Col*, or *Sha*/*Col* alleles of *KCS18* (QTL allele/gene allele). For each of the four combinations, a pool of 100 F1 seeds obtained from 10 independent crosses was phenotyped for CLR. Seeds carrying the *Col* allele of *KCS18* presented a nearly similar CLR regardless of the presence of the *Bay* or *Sha* allele at the QTL (Fig. 4). Since the *kcs18-1* and *Col* alleles are semi dominant (our observation and [37]), seeds carrying *Bay*/*kcs18-1* have a reduced LEN ratio compared to seeds carrying *Bay*/*Col*. However, a significant difference in CLR was observed when *Bay* and *Sha* were associated with the knock-out *kcs18-1* allele, with plants carrying the *Sha* allele displaying a reduced CLR compared to plants carrying the *Bay* allele (Fig. 4), in agreement with our previous results. Furthermore, an ANOVA showed a significant *CLR.2* genotype x *KCS18* allele interaction (*p*<0.1%), confirming that *CLR.2* and the *KCS18* gene either interact or are identical.

All together the mapping data, the T-DNA mutant analysis and the quantitative complementation test lead us to conclude that *CLR.2* is *KCS18*, although we cannot totally exclude at this stage that KCS17 is involved.

### A Single Amino Acid Substitution in KCS18 Protein Associated with CLR Reduction

**Figure 5:**
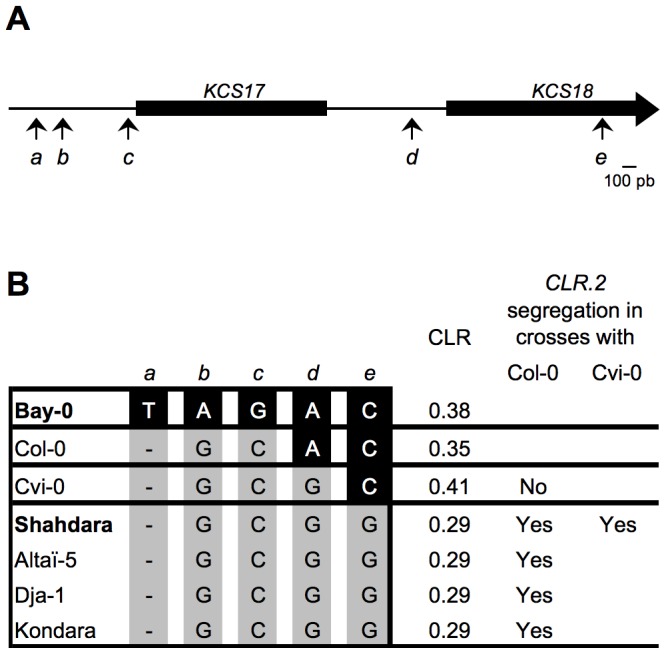
One SNP in *KCS18* coding sequence is sufficient to modulate chain length ratio. A, Promoters and coding sequences of the *KCS17* and *KCS18* genes. SNPs identified between Bay-0 and Shahdara are indicated with letters and their respective positions are indicated by arrows. B, Regions covering SNPs shown in A have been sequenced in the indicated accessions. The nucleotide for each SNP is shown for all accessions. CLR values are indicated at the right in addition to *CLR.2* segregation in crosses with Col-0 or Cvi-0.

To identify the causal polymorphism(s) responsible for the CLR difference observed between Bay-0 and Shahdara, we sequenced the Bay and Sha alleles of *KCS17* and *KCS18* (promoters and coding regions). Five Single Nucleotide Polymorphism (SNP) differences were found in that region (named *a* to *e*, Fig. 5A and 5B), with one, *e*, resulting in a L407V amino acid change in the coding sequence of *KCS18*. This leucine residue is highly conserved across proteins and taxa. Indeed, out of 100 homologs retrieved through a protein-BLAST search using the Bay-0 KCS18 protein sequence in the land plant database [38], all 100 shared the Bay-0 state (leucine) at KCS18 position 407 (data not shown). To assess the importance of this leucine 407 in the coding sequence of *KCS18* gene, a dCAPS allowing to genotype the *e* SNP was designed and 568 Arabidopsis accessions originating from the whole species range were screened. Thirty-five had a Sha-like genotype (Table S2). Twenty-one of these 35 accessions were phenotyped for CLR and all displayed a low CLR, similar to Shahdara (Table S2). This perfect association indicates that this polymorphism could be responsible for the difference in CLR observed between Bay and Sha alleles.

To further analyze if the *a*, *b*, *c* and *d* polymorphisms could also be involved in the CLR phenotype, we sequenced the region spanning the *KCS17* and *KCS18* genes in three representative accessions sharing the Sha genotype at the *e* SNP and displaying a low CLR as in Shahdara (i.e. Altaï-5, Dja-1 and Kondara) and in Col-0. All Sha-like accessions shared the same haplotype whereas Col-0 displayed an intermediate haplotype between Bay-0 and Shahdara (Fig. 5B), suggesting that *a*, *b* and c SNPs were not responsible, or at the least not sufficient, for the CLR variation observed. This association was confirmed by analyzing F2 individuals issued from three crosses between Sha-like accessions and Col-0 (Altaï-5 x Col-0, Dja-1 x Col-0 and Kondara x Col-0) and RILs derived from a Shahdara x Col-0 cross. The CLR phenotype segregated in these populations with the *CLR.2* region, whereas the *a*, *b* and *c* SNPs did not (Fig. 5B and supplemental Fig. S3), demonstrating that these SNPs were not responsible for the CLR variation observed. Two additional populations segregating either for the *d* SNP alone (i.e. a Cvi-0 x Col-0 RIL set) or for the *e* SNP alone (i.e. an F2 population from a cross between Shahdara and Cvi-0) were analyzed (Fig. 5B). These populations showed that CLR did not segregate among the Cvi-0 x Col-0 RILs, whereas segregation was observed in the Shahdara x Cvi-0 F2s (Supplemental Fig. S3), demonstrating that the *e* but not the *d* SNP was responsible for the CLR difference observed at *CLR.2*. A contribution from polymorphisms outside of the candidate interval in some accessions cannot be completely ruled out, but this is very unlikely. Overall, these genetic analyses established that *a*, *b*, *c* and *d* SNPs, which are located in the promoter regions of *KCS17* and *KCS18*, were not involved in the CLR phenotype. In accordance with that, no significant difference in *KCS17* and *KCS18* expression levels could be observed between developing seeds of arHIF196_[Bay]_ and arHIF196_[Sha]_ (data not shown). Thus, the unique L407V amino acid change in the Shahdara KCS18 protein represented the causative quantitative trait nucleotide (QTN) for *CLR.2*.

### KCS18 Bay and Sha Protein Isoforms Showed Different FA Elongation Activities

**Figure 6:**
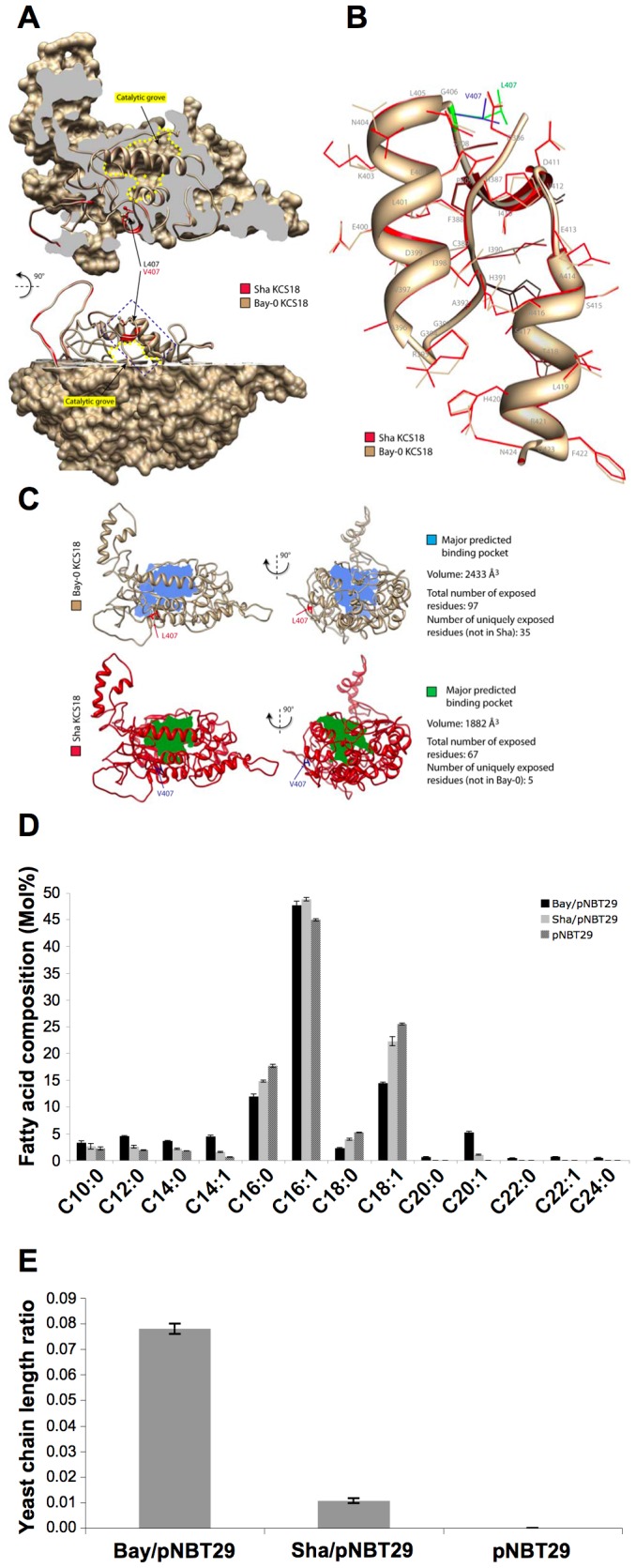
A single amino acid change modulates KCS18 activity. A, General view of Bay and Sha KCS18 protein models superimposition. Sha KCS18 is shown in red as a ribbon model, Bay KCS18 is shown in tan as a ribbon and surface model. Bay KCS18 model surface is clipped to reveal the position of the predicted catalytic grove, circled in yellow, and the position of the region harboring conformational changes. In both models, the side chain of the variable residue (407) is shown. The dotted blue box delimits the region highlighted in B. B, A close view of the region harboring conformational changes between Sha (red) and Bay (tan) KCS18 models. The models are depicted as ribbons with residue side chain backbones shown. Residue 407 is shown in blue (Sha model) and green (Bay model). C, Analysis of predicted binding pockets in Sha and Bay KCS18 models. Models are shown as ribbons in tan (Bay) and red (Sha), with residue 407 highlighted. The major binding pocket predicted by PocketFinder is shown as a filled volume in blue (Bay) and green (Sha). D, Fatty acid composition of yeast cells transformed with an empty vector (pNBT29), or a vector harboring the Bay (Bay/pNBT29) or Sha (Sha/pNBT29) *KCS18* coding sequence. Values are a mean of 6 samples (3 replicates, 2 independent transformations). Bars represent SE. E, Yeast chain length ratio (the sum of all 20- 22- and 24-carbon fatty acids divided by the sum of all 10- to 18-carbon fatty acids) was measured on yeast cells expressing Bay (Bay/pNBT29), Sha (Sha/pNBT29) or no KCS18 protein (pNBT29).

The conservative amino acid change leucine to valine corresponds to the addition of a CH2-group on the side chain of the residue. To gain insight into the impact of the L407V change on KCS18 enzyme activity, the L407V substitution was simulated on the model structure generated by Joubès et al. [39]. Residue 407 sits close to the predicted catalytic grove accommodating acyl-CoA substrates but with no access to this catalytic grove (Fig. 6A). The L407V substitution was not predicted to cause a major alteration in the KCS18 model structure. However, it is predicted to cause local conformational changes due to re-arrangements of amino acid side chains between residues 409 and 417 (Fig. 6A and 6B). Analysis of binding pockets with PocketFinder [40] indicated that in the Sha KCS18 model, the volume of the catalytic grove is reduced by 25% compared to the Bay model, with 40 residues changing exposure to this grove (Fig. 6C). This could result to a reduced capacity of Sha KCS18 enzyme to elongate long chain fatty acid.

To compare the activity between Bay-0 and Sha KCS18, the two KCS18 elongase isoforms were expressed in yeast cells. Yeast cells expressing Bay or Sha KCS18 protein were able to produce VLCFAs, whereas fatty acids with more than 18 carbons were not detected in yeast cells transformed with the empty vector pNBT29 (Fig. 6D), demonstrating that the KCS elongases from both plant accessions were enzymatically active with endogenous yeast fatty acid substrates. In cells expressing a comparable level of the two KCS18 isoforms (Supplemental Fig. S4), those cells expressing the Sha protein displayed a lower CLR than cells expressing the Bay protein (Fig. 6E). This was the result of a reduced level of C20:0 and C20:1 and an increased level of C18:0 and C18:1 in cells expressing the Sha KCS18 isoform compared to cells expressing the Bay isoform (Fig. 6D). This suggested that the Sha KCS18 isoform used less efficiently C18 fatty acid substrates to synthesize VLCFAs than the Bay-0 isoform.

## Discussion

The aim of this study was to identify genetic factors governing natural variation in VLCFA content by a QTL analysis using an *Arabidopsis thaliana* RIL population derived from a cross between the accessions Bay-0 and Shadhara. Two QTL involved in VLCFA content were identified, one of which, *CLR.2*, had a major effect and explained 77% of the total genetic variation.

### *KCS18* is a Major Control Point for VLCFA Synthesis in Plants

Fine mapping of *CLR.2* led us to an 18 kb region containing the *KCS17* and *KCS18* genes, encoding for β-ketoacyl-CoA synthase proteins, which both represented good candidates for *CLR.2*. Indeed, VLCFAs are synthesized by an endoplasmic reticulum membrane-bound fatty acid elongation complex, composed of four enzymes, the first one being a β-ketoacyl-CoA synthase (KCS) [11], [12]. In Arabidopsis, 21 genes encode for KCS proteins [41], whereas only one or two genes encode for each of the three other enzymes of the complex. This multiplicity of *KCS* genes, which have organ- and tissue-specific expression [39], suggested that KCS enzymes determine the substrate and tissue specificities of fatty acid elongation. Only three *KCS* genes (*KCS7* and our two candidate genes, *KCS17* and *KCS18*) are expressed specifically in flowers and siliques [39], [42], [43], reinforcing the hypothesis that they could play a role in seed lipid metabolism. Study of *kcs17* and *kcs18* T-DNA insertion mutants indicated that *KCS18* played an important role in CLR and showed that *KCS17* likely was not involved in the CLR phenotype (Fig. 3C), suggesting that variations in *KCS18* could explain *CLR.2*. This hypothesis was confirmed by our quantitative complementation test.

Previous studies of Arabidopsis VLCFA-deficient EMS-mutants (named *fae1* for fatty acid elongation) showed that the *KCS18* gene product was required in the seed for the elongation of C22:1 (erucic acid) from C18:1 [37], [44], [45]. Similarly in rapeseed, it was shown that two genes homologous to Arabidopsis *KCS18* were involved in erucic acid synthesis [46], [47] and that the near absence of erucic acid in LEAR cultivars was caused by loss-of-function mutations in *KCS18* genes [48], [49]. Our results converged to show that *KCS18* is the quantitative gene responsible for *CLR.2* phenotypic effect. To our knowledge, our study is the first example of a QTL involved in seed lipid metabolism that was cloned down to the gene level in Arabidopsis.

### A Single Amino Acid Change is Responsible for Variable VLCFA Content in Natural Plant Populations

Having identified the gene responsible for *CLR.2*, we examined the molecular mechanisms underlying this QTL effect. Sequencing of the promoters and coding regions of *KCS17* and *KCS18* in Bay-0 and Shahdara revealed five SNPs between both accessions. Phenotype-genotype association allowed us to exclude four of these five SNPs and culminated with the identification of the quantitative trait nucleotide (QTN) responsible for the CLR difference observed between Bay-0 and Shahdara. The association approach revealed that all the accessions displaying a low CLR came from the same geographical area in Central Asia (Table S2), a correlation already noticed by Millar and Kunst [50] and O'Neill et al. [30]. This geographic isolation suggested that having a hypomorphic allele of *KCS18* could either provide an adaptive advantage in that region or result from a recently emerged mutation that is rapidly (but possibly neutrally) expanding among these populations. This raises the question about the role of VLCFAs in seed oils. Indeed, VLCFAs are not widely distributed in seeds of higher plants, with the exception of Brassicaceae species and some other species like meadowfoam (*Limnanthes alba*) or Nasturtium (*Tropaeolum majus*). In Arabidopsis, examination of the seed fatty acid composition of 100 accessions showed that all contained a significant proportion of VLCFAs, averaging 31% (w/w) of total seed fatty acids [50]. This consistent and widespread high level of VLCFAs argues that efficient VLCFA synthesis might give the plant a selective advantage. By contrast, LEAR cultivars with nearly no VLCFAs in their seed oil do not show significant growth changes in the field. To date, no exact physiological or biochemical role of VLCFAs in seed fatty acid composition has been shown. However, Boyes et al. [51] revealed that one Arabidopsis *kcs18* EMS-mutant (*fae1-1*) had subtle modified vegetative development with slow leaf growth and early flowering transition. Indeed, using a high-throughput phenotyping platform (“The Phenoscope”, http://www.inra.fr/vast/projects.htm), we were able to confirm the reduced rosette leaf growth in the *fae1-1* KCS18 mutant and extended this observation to an additional T-DNA mutant allele, *kcs18-1* (data not shown) compared to wild type in standard growing conditions. However, using the same phenotyping platform, no difference was observed between arHIF196_[Bay]_ and arHIF196_[Sha]_. This is likely explained by the fact that both *kcs18-1* and *fae1-1* display a strongly reduced CLR compared to wild type (CLR_mutants_ = 0.01, CLR_wt_ = 0.35, this work and data not shown) whereas arHIF196_[Sha]_ shows only a slightly reduced CLR compared to arHIF196_[Bay]_ (0.30 versus 0.37). This slight difference may not be sufficient to lead to a differential plant growth under our conditions.

The identification of a second hypomorphic allele of *KCS18* in the Kyr-1 accession collected from the same geographical area (data not shown) reinforces the hypothesis that a low CLR may bring a selective advantage in that region. However, more experiments will be needed to conclude about this possible adaptive response.

### Natural Variation Reveals a New KCS18 Isoform with Altered Activity

In this study, we identified the QTN responsible for the *CLR.2* phenotype. This nucleotide corresponds to the amino acid 407 in KCS18 protein, a leucine in Bay-0 and a valine in Shahdara. Previous studies in Arabidopsis and Brassica identified cysteine 223 (C223) [52], asparagine 424 (N424), histidine 391 (H391) [53] and serine 282 (S282) [54], [55] as important residues for KCS18 activity. Cysteine 223, H391 and N424 constitute the KCS18 putative catalytic triad [52], and S282 was proposed to form a key transition between an hydrophobic and an hydrophilic region of a putative catalytic domain spanning from valine 267 to glycine 365 [54]. All mutation tested at positions C233 and H391 nearly abolished KCS18 activity [52], [53], whereas mutations at N424 and S282 either abolished KCS18 activity or only slightly modulated its activity [53], [54]. Contrasting with previously analyzed residues, the QTN reported here is located outside of the KCS18 catalytic domain. However this QTN encodes an amino acid strongly conserved in plants, suggesting that this residue is important for KCS18 activity. Consistently, the resulting leucine to valine exchange, a conserved amino acid substitution, is sufficient to impair KCS18 enzymatic activity in plant and yeast. Because VLCFA synthesis is very important to the plant physiology [56], it is likely that non conserved amino acid substitutions that would more drastically alter KCS18 protein structure and function have been selected against. By altering residue spatial organization rather than physico-chemical properties, substitutions affecting the elongase activity remotely from the catalytic domain, such as L407V, offer the opportunity to finely modulate KCS18 properties without drastically impairing its catalytic activity. Here, the study of natural variation gave rapid access to the molecular bases of a subtle biochemical phenotype. Thus, this work illustrates QTL analysis in Arabidopsis as an advantageous tool to identify naturally occurring functional alleles, which could be used for the fine engineering of oil production.

Rapeseed is a major crop worldwide for oil production. Even though the seed oil production has been greatly enhanced and the panel of seed fatty composition widely enriched largely due to breeding efforts, there is an increasing need to improve these traits. One way to progress is to expand the available variation present within the existing gene pool. As *KCS18* genes were shown to be involved in erucic acid content in rapeseed [46], [47], experiments to isolate new *KCS18* alleles by tilling and ecotilling have been undertaken [57], [58]. Approximately 1,350 M2 *Brassica napus* plants and 117 accessions from three different Brassica species (i.e *B. rapa*, *B. oleracea* and *B. napus*) were screened, respectively, in both studies. These screens allowed the identification of four new *KCS18* alleles, which, in addition to Arabidopsis allele identified in this study, will serve as important genetic resources to develop new LEAR cultivars by breeding.

### Conclusion

Using a RIL population obtained from a cross between the *Arabidopsis thaliana* accessions Bay-0 and Shahdara, two QTL involved in seed VLCFA content have been identified and one major QTL, named *CLR.2* was cloned down to the gene level. To our knowledge, this is the first time that a QTL involved in seed lipid metabolism has been cloned in Arabidopsis. *KCS18* is the gene responsible for the *CLR.2* QTL and the precise QTN responsible for the CLR phenotype was identified, revealing a new allele of *KCS18*. Even though the reaction catalyzed by the *KCS18* elongase during VLCFA synthesis in Arabidopsis was already known, the discovery of this new allele mapped an additional residue important for KCS18 activity. Finally, this study highlights the potential of QTL analyses to discover unknown but naturally occurring alleles of a gene, which, in this case, could be of great interest to oil crop breeding as a reservoir for natural variants in order to alter VLCFA composition.

## Materials and Methods

### Plants Material and Growth Conditions

The *Arabidopsis thaliana* Bay-0 x Shahdara RIL population was obtained from the Versailles Biological Resource Centre for Arabidopsis (http://dbsgap.versailles.inra.fr/vnat/). For this work, we have used a set of 164 F7 genotyped RILs and the 69-microsatellite marker map (“Map2”).

HIFs to confirm the *CLR.2* QTL were obtained from the Versailles Biological Resource Centre for Arabidopsis. HIFs were developed from individual F7 RILs that still segregated in a limited genomic region [33], [34]. For each RIL, several plants were sown and genotyped individually for the appropriate markers across the segregating region and three independently fixed plants for each allele (composing the HIF) were chosen and allowed to self-fertilize. F8 seeds from these homozygous plants were then phenotyped as described below to identify the phenotypic consequences of Bay versus Sha alleles at the segregating region.

All other accessions, RILs and F2 populations used in this work were ordered from the Versailles Biological Resource Centre for Arabidopsis (http://dbsgap.versailles.inra.fr/vnat/).

The T-DNA insertion mutants in *At4g34510* (*kcs17-1* and *kcs17-2*) and *At4g34520* (*kcs18-1* and *kcs18-2*) were ordered from the Nottingham Arabidopsis Stock Centre (NASC, http://arabidopsis.info/) as N318935, N871890, N108732 and N586589 respectively. The four mutants are in a Col-0 background.

For CLR phenotyping, plants were grown in growth chamber under long-day conditions (16/8 hr photoperiod at 150 µmol photons m^−2^ s^−1^); day temperature was 21°C and night temperature was 18°C, hygrometry 65%. Screen for recombinants was performed in greenhouse in September under natural light supplemented with sodium lamps to provide a 16 h photoperiod. Temperature was 21°C during the day, 18°C during the night.

### Fatty Acid Analysis

Total seed and yeast fatty acid content and fatty acid composition were determined by direct transmethylation followed by gas chromatography and then flame ionization detection (GC-FID) [59], [60]. Quantification of fatty acids was done by comparison to the methyl heptadecanoate issued from the heptadecanoic acid C17:0 (H-3500, Sigma-Aldrich), added to each sample as an internal standard. Fatty acids are expressed as % of the sum of all fatty acids. We define the “fatty acid chain length ratio” (CLR) as the sum of areas under peaks for all 20–22 and 24-carbon fatty acids divided by the sum of areas under peaks for all 16- and 18-carbon fatty acids for seeds and as the sum of areas under peaks for all 20–22 and 24-carbon fatty acids divided by the sum of areas under peaks for all 10- and 18-carbon fatty acids for yeast cells.

100 seeds were counted with a seed counter (elmor C3, ELMOR, Switzerland) and dried overnight at 105°C into a screw-capped centrifuge glass tube. A 3-mL volume of transmethylation solution (methanol:sulfuric acid:toluene:C17:0 at 5 µg/µL in 1:0.025:0.3:0.002 v/v/v/v) were added and samples were incubated at 95°C for 1.5 h. After cooling on ice, 1.2 mL of hexane and 2.3 mL of bidistilled H2O containing 0.9% NaCl were added. The tubes were vigorously shaken followed by centrifugation at 2000 rpm for 5 min. Fatty acid methyl esters were extracted in the upper hexane layer and an aliquot of 1 µL of the organic phase was analyzed by GC on a 30 m×0.53 mm EC™-WAX column (Alltech Associates Inc., Deerfield, USA) and quantified using a flame ionization detector. The gas chromatograph was programmed for an initial temperature of 160°C for 1 min, followed by an increase of 20°C min^−1^ to 190°C and a ramp of 4°C min^−1^ to 230°C, with a 9-min hold of the final temperature.

For QTL detection, only 15 mature F7 seeds from each of the 164 RILs were analyzed with 1.32 mL of transmethylation solution, 500 µl of hexane and 1 mL of 0.9% NaCl. The organic phase was half concentrated under nitrogen gas and 1 µL was analyzed. Each sample was analyzed in triplicate.

Yeast cells (corresponding to 25 mg dry weight) were collected by centrifugation, washed with water and freeze-dried for 72 h. The pellet was disrupted by vortexing in the presence of 0.45 mm glass beads, 200 µg of C17:0 and 2 mL of 2.5% (v/v) sulfuric acid in methanol. The samples were heated for 90 min at 80°C. Fatty acid methyl esters (FAME) were extracted by addition of 3 mL of NaCl 0.9%, 0.9 mL of hexane and with vigorous shaking. Samples of the organic upper phase were separated by GC using a 7890A chromatograph (Agilent) with a Factor Four VF-23 ms 30 m×0.25 mm capillary column (Agilent). The carrier gas was helium at an inlet pressure of 1 mL min^−1^. The column temperature program started at 40°C for 1 min, ramping to 120°C at 40°C min^−1^, holding for 1 min at 120°C, ramping to 210°C at 3°C min^−1^ and holding for 10 min at 210°C.

### QTL Mapping

QTL analyses were performed using the Unix version of QTLCartographer 1.14 [61], [62] and standard methods for interval mapping (IM) and composite-interval mapping (CIM) [63]. First, IM was carried out to determine putative QTL involved in the variation of the trait, and then CIM model 6 of QTLCartographer was performed on the same data: the closest marker to each local LOD score peak (putative QTL) was used as a cofactor to control the genetic background while testing at another genomic position. The walking speed chosen for QTL analysis was 0.1 cM. The global LOD significance threshold (2.3 LOD) was estimated from 1000-permutations test analysis, as suggested by Churchill and Doerge [64]. Additive effects of detected QTL were estimated from CIM results, as representing the mean effect of the replacement of the Sha alleles by Bay alleles at the locus. The contribution of each identified QTL to the total phenotypic variation (*R* ^2^) was estimated by variance component analysis, using phenotypic values for each RIL. The model used the genotype at the closest marker to the corresponding detected QTL as random factors in ANOVA, performed using the *aov* function in R. Only homozygous genotypes were included in the ANOVA analysis.

### Fine Mapping

CLR phenotyping for the confirmation of the QTL segregation during the fine mapping process was performed as described above. Three HIFs (048, 057 and 196) were used to confirm the *CLR.2* locus. For each HIF, three individuals fixed for each parental allele were phenotyped to confirm the relative effect of Bay and Sha alleles on CLR. For the chosen HIF (HIF196), 384 F9 plants were genotypically screened to identify recombinants (rHIFs) within the segregating interval. Genotyping involved microsatellites or indel markers to identify recombination events within the candidate region. Once recombinants had been identified, dCAPS markers and direct sequencing were used to refine and localize recombination breakpoints to smaller intervals when needed. Informative rHIFs were then tested for the segregation of the CLR phenotype by fixed-progeny testing: for each rHIF, 24 plants were grown and genotyped to isolate individuals fixed for the parental alleles at the remaining heterozygous interval. Three plants fixed for each parental allele were then phenotyped for the CLR.

Advanced rHIF line 196 (arHIF196) that segregates solely for a 22 kb region (including the candidate region) was obtained from a cross between two different rHIFs lines (rHIF196-55 and rHIF196-75; Fig. 2A) with adequate genotypes (rHIFs recombined immediately to the north or immediately to the south of the *CLR.2* final interval and with adequate genotype elsewhere), as described by Loudet et al. [35].

### Genotyping Mutants and Alleles by PCR

All sequence primers are described in Table S3.

Primers used for genotyping SNPs were designed using dCAPS Finder 2.0 (http://helix.wustl.edu/dcaps/dcaps.html, [65]). For the *d* SNP, a 180-bp fragment was amplified with primers dCAPS4.16493-F and dCAPS4.16493-R2 and digested with NdeI. Only the fragment originating from the Bay fragment was digested into 158- and 22-bp fragments. For the *e* SNP, a 222-bp fragment was amplified with primers dCAPS4.16495-F and dCAPS4.16495-R and digested with BsuRI. Only the fragment originating from the Bay allele was digested into 202- and 20-bp fragments.

For *kcs17-1*, wild type allele was genotyped with KCS17-F1′ and KCS17-R1′, while mutant allele was genotyped with KCS17-R1’ and GABI_o8409. For *kc17-2*, wild type allele was genotyped with KCS17-F2 and KCS17-R3, while mutant allele was genotyped with KCS17-F2 and LB3-Sail. For *kcs18-1* and *kc18-2* mutants, wild type allele was genotyped with KCS18-F1 and KCS18-R1, while mutant alleles were genotyped with KCS18-R1 and JIC-SM-LB2 for *kcs18-1* and KCS18-F1 and LB-Salk2 for *kcs18-2*.

### RT-PCR

Three plants per genotype (Col-0, *kcs17-1*, *kcs17-2*, *kcs18-1* and *kcs18-2*) were grown in growth chamber under long day condition (see above). When flowering, five flowers at pollination stage per plant were labeled 10-days prior to silique harvesting. 15 siliques per genotype were then dissected to isolate 10-days-old seeds. These seeds were pooled and total RNA was extracted and DNAse-treated using the RNeasy Plant Mini Kit (Qiagen) following the manufacturer’s protocol. 250 ng of total RNA was reverse transcribed by the RevertAid M-MuLV Reverse Transcriptase (Fermentas) with an oligo (dT) primer according to the manufacturer’s protocol. cDNA were diluted 10 times and 1 µL were used as template in a 20 µL PCR reaction.

PCR primers specific for *KCS17* (KCS17-For3 and KCS17-Rev1), *KCS18* (KCS18-For1 and KCS18-Rev1) and *APT* (APT-RT1 and APT-RT2) amplified products of 1635 bp, 1544 bp and 554 respectively. The amplification conditions were 94°C (30 s), 52°C (30 s), and 72°C (1.30 min), and the number of cycles was 25, 35 and 40 for *APT*, *KCS18* and *KCS17* respectively. All sequence primers are described in Table S3.

### Quantitative Complementation

For the quantitative complementation assay, F1 seeds were generated by crossing arHIF196_[Bay]_ to *kcs18-1*, arHIF196_[Sha]_ to *kcs18-1*, arHIF196_[Bay]_ to Col-0 and arHIF196_[Sha]_ to Col-0. For each combination, 10 independent reciprocal crosses (five in one direction, five in the reverse direction) were performed. For each combination, 100 F1 seeds were then used directly for CLR phenotyping. To control the effectiveness of the crosses, 5 F1 seeds per combination were germinated on filter paper and seedlings were genotyped for both the gene allele (with KCS18-F1/KCS18-R1 for the wild type allele and KCS18-R1/JIC-SM-LB2 for the mutant allele) and the QTL allele (with dCAPS4.16495-F and dCAPS4.16495-R).

### Protein Model Structure Analysis

KCS18 protein model for the Bay isoform is described in Joubès et al. [39]. KCS18 protein model for the Sha isoform was generated based on Bay isoform model using Modeller 9v7 [66]. Models were rendered using the UCSF Chimera software [67], and structures matched using the MatchMaker tool in Chimera. Binding pocket volumes and exposed residues were predicted using the PocketFinder server [40].

### Plasmid Construction

For expression in yeast, *KCS18* gene from Bay-0 or Shahdara were cloned into pNBT29 plasmid [68]. For this purpose, the coding sequence of *KCS18* was amplified by PCR using a proofreading polymerase and the forward primer KCS18-For1 and the reverse primer KCS18-Rev5 (Table S3). This DNA fragment was then digested with BglII and cloned between the SmaI and BglII sites of pNBT29 resulting in *KCS18* gene in frame with the GFP. These AtKCS18_Bay_/pNBT29 and AtKCS18_Sha_/pNBT29 vectors allowed inducible expression of GFP-tagged proteins when yeasts were grown in galactose-containing medium.

### Yeast Strain and Growth Conditions

The haploid strain *S*. *cerevisiae* BY4741 (MATa; his3Δ 1; leu2Δ 0; met15Δ 0; ura3Δ 0) (EUROSCARF) was used in this study. Plasmids were introduced into yeast using the lithium acetate method [69]. Transformed strains were grown on synthetic media containing 0.67% yeast nitrogen base (without amino acids) (BD bioscience), 2% sugar (glucose or galactose), and 0.02% casaminoacids. For all experiments, cultures were performed at 28°C with shaking at 200 rpm.

### Total Yeast Extracts and Immunoblot

After growth in galactose media for approximately 16 h, cells (corresponding to 0.75 mg dry weight) were harvested, resuspended into a final volume of 500 µL, and total protein extracts were prepared using the NaOH/trichloroacetic acid (TCA) lysis technique as described by Volland et al. [70]. Fifty microliters of 1.85 M NaOH was added to the sample and cells were disrupted by vortexing and incubating on ice for 10 min. Proteins were precipitated 10 min on ice by adding 50 µL of 50% TCA. The resulting protein pellet was resuspended in 50 µL of loading buffer containing two volumes of 2× sample buffer (100 mM Tris-HCl, pH 6.8, 4 mM EDTA, 4% SDS, 20% glycerol and bromophenol blue), one volume of Tris base 1 M and 2% mercaptoethanol.

Proteins were separated by SDS-PAGE using ready-to-use NuPage Novex 4–12% Bis–Tris gels and NuPAGE MES SDS running buffer (Invitrogen, Cergy Pontoise, France). For immunoblotting experiments, proteins were transferred onto Immobilon-P PVDF membranes (Millipore, Saint Quentin en Yvelines, France) and probed with monoclonal antibodies raised against GFP at 1∶2000 (Roche Diagnostics) and actin at 1∶1000 (Abcam). Primary antibodies were detected using horseradish peroxidase-conjugated anti-rabbit or anti-mouse immunoglobulin G secondary antibodies and revealed using SuperSignal West Dura Extended Duration Substrate (Perbio, Brebières, France). Luminescence from peroxidase activity was recorded using the LAS-3000 imaging system and MULTIGAUGE software from Fujifilm (Saint Quentin en Yvelines, France).

## Supporting Information

Figure S1 **Fatty acid composition and CLR phenotype of Bay-0 and Shahdara accessions.** A, Fatty acid composition of Bay-0 and Shahdara accessions. Bars represent SE values (n = 4, 2 repetitions were done on 2 plants). B, Comparison of CLR for Bay-0 and Shahdara accessions. CLR was determined from 100 seeds per plants. Bars represent SE values (n = 4, 2 repetitions were done on 2 plants). Significance in t-test, *** p<10^−5^.
(TIF)

Figure S2 **Frequency distribution of the chain length ratio in Bay-0 x Shahdara RILs.** Frequency distribution of the mean chain length ratio for 164 RILs (n = 3). The arrows depict the mean values of the parental lines (S, Shahdara; B, Bay-0). RILs, recombinant inbred lines.
(TIF)

Figure S3 ***a*** **,** ***b, c*** **and** ***d*** **SNPs are not involved in** ***CLR.2*** **phenotype segregation.** Phenotypic effect of the allele present at *CLR.2* as tested in different segregating populations. Sha-like alleles (light grey) and Cvi allele (dark grey) are tested against Col allele (black) in the mentioned crosses. Finally, Sha and Cvi alleles are tested in the last cross. CLR was determined from 10 plants per *CLR.2* genotype (100 seeds per plant). Error bars represent SE values (n = 10). Significance in t-test, ***p<10^−6^.
(TIF)

Figure S4 **Yeast cells transformed with Bay/pNBT29 or Sha/pNBT29 express a comparable level of the two KCS18 isoforms.** A, Western blot on protein extracts from yeast cells expressing Bay/pNBT29 or Sha/pNBT29 was performed using antibodies raised against GFP and actin as control. Three replicates were probed for each protein extracts. B, Comparison of the relative KCS18 protein quantity measured from the luminescence of the peroxidase activity.
(TIF)

Table S1 **List of genes in the** ***CLR.2*** **18-kb candidate interval.**
(TIF)

Table S2 **List of Sha-like accessions.** The name and the AV code of the accessions are mentioned with the country and city/region where they have been collected. nd, not determined. A low CLR phenotype means ≤0.33.
(TIF)

Table S3 **List of primers used.**
(TIF)
