# Supplementary figures and images for: Natural Variation in Seed Very Long Chain Fatty Acid Content Is Controlled by a New Isoform of KCS18 in Arabidopsis thaliana
Source: PLoS One. 2012 Nov 8;7(11):e49261. doi: 10.1371/journal.pone.0049261 (PMC3493540; doi:10.1371/journal.pone.0049261)

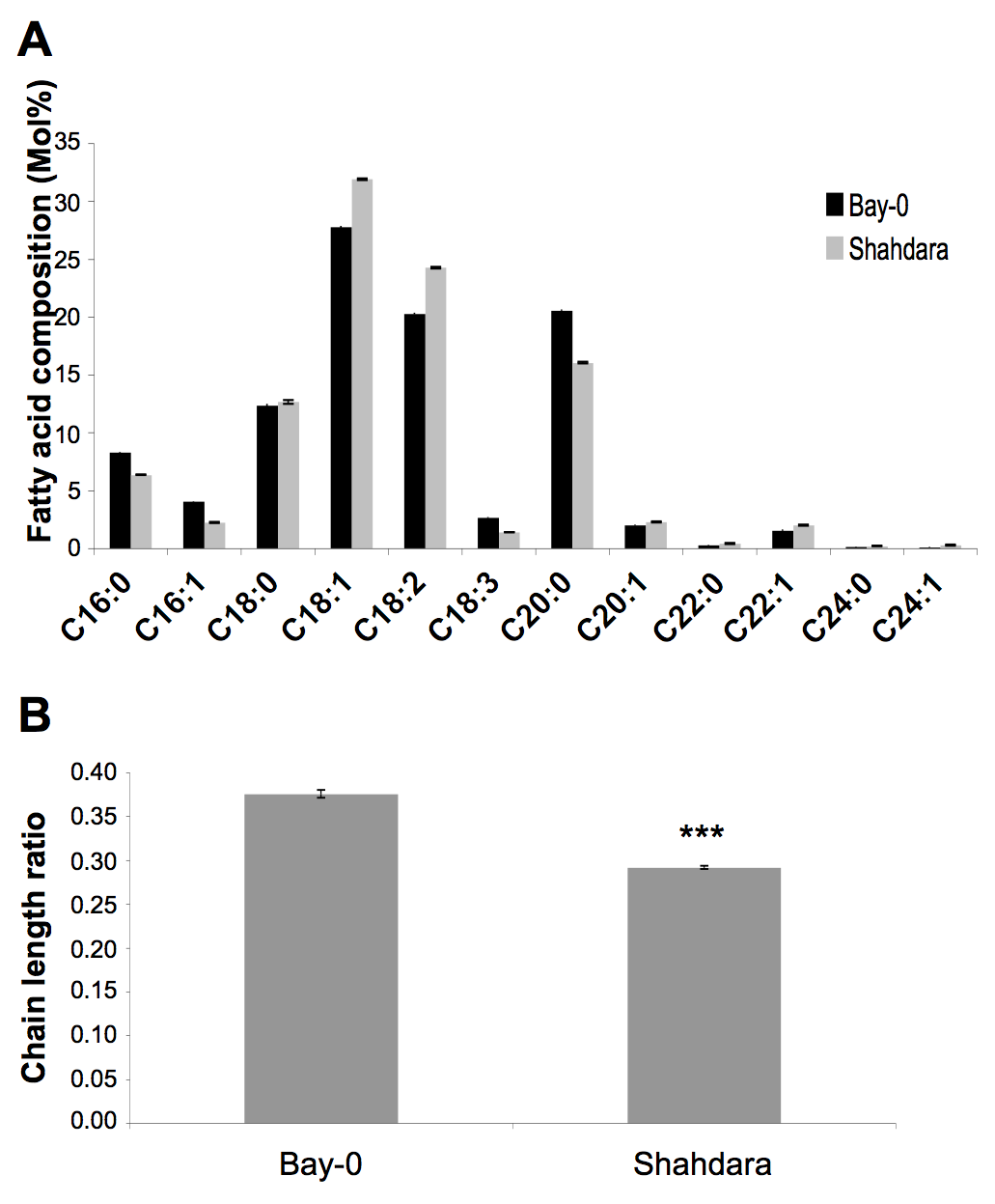

Supplement: Figure S1 — Fatty acid composition and CLR phenotype of Bay-0 and Shahdara accessions. A, Fatty acid composition of Bay-0 and Shahdara accessions. Bars represent SE values (n = 4, 2 repetitions were done on 2 plants). B, Comparison of CLR for Bay-0 and Shahdara accessions. CLR was determined from 100 seeds per plants. Bars represent SE values (n = 4, 2 repetitions were done on 2 plants). Significance in t-test, *** p<10−5. (TIF) [file pone.0049261.s001.tif]

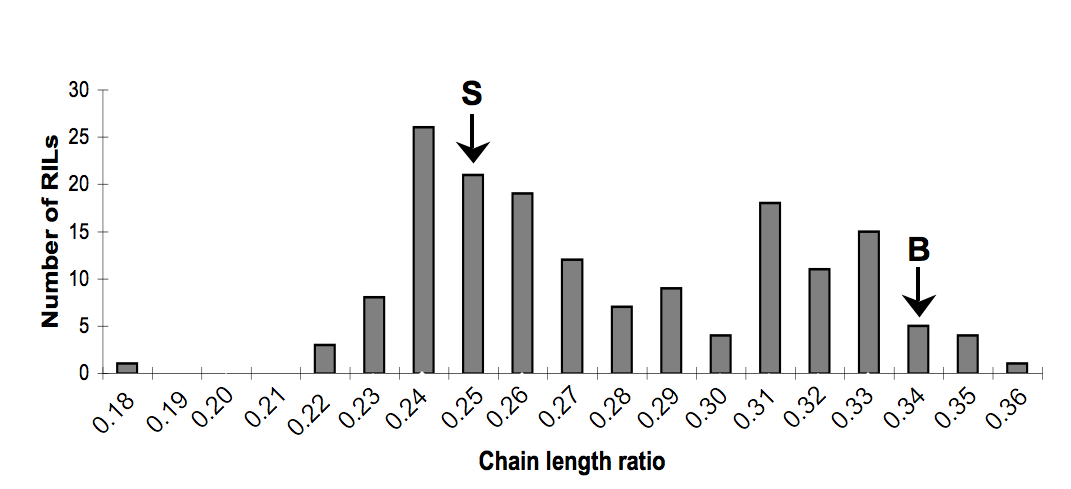

Supplement: Figure S2 — Frequency distribution of the chain length ratio in Bay-0 x Shahdara RILs. Frequency distribution of the mean chain length ratio for 164 RILs (n = 3). The arrows depict the mean values of the parental lines (S, Shahdara; B, Bay-0). RILs, recombinant inbred lines. (TIF) [file pone.0049261.s002.tif]

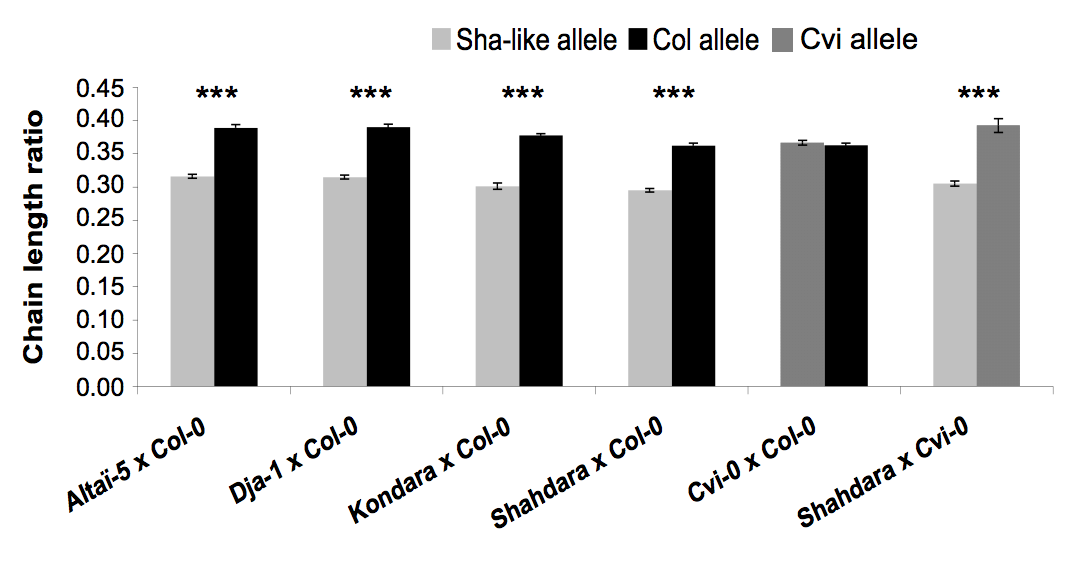

Supplement: Figure S3 — a , b, c and d SNPs are not involved in CLR.2 phenotype segregation. Phenotypic effect of the allele present at CLR.2 as tested in different segregating populations. Sha-like alleles (light grey) and Cvi allele (dark grey) are tested against Col allele (black) in the mentioned crosses. Finally, Sha and Cvi alleles are tested in the last cross. CLR was determined from 10 plants per CLR.2 genotype (100 seeds per plant). Error bars represent SE values (n = 10). Significance in t-test, ***p<10−6. (TIF) [file pone.0049261.s003.tif]

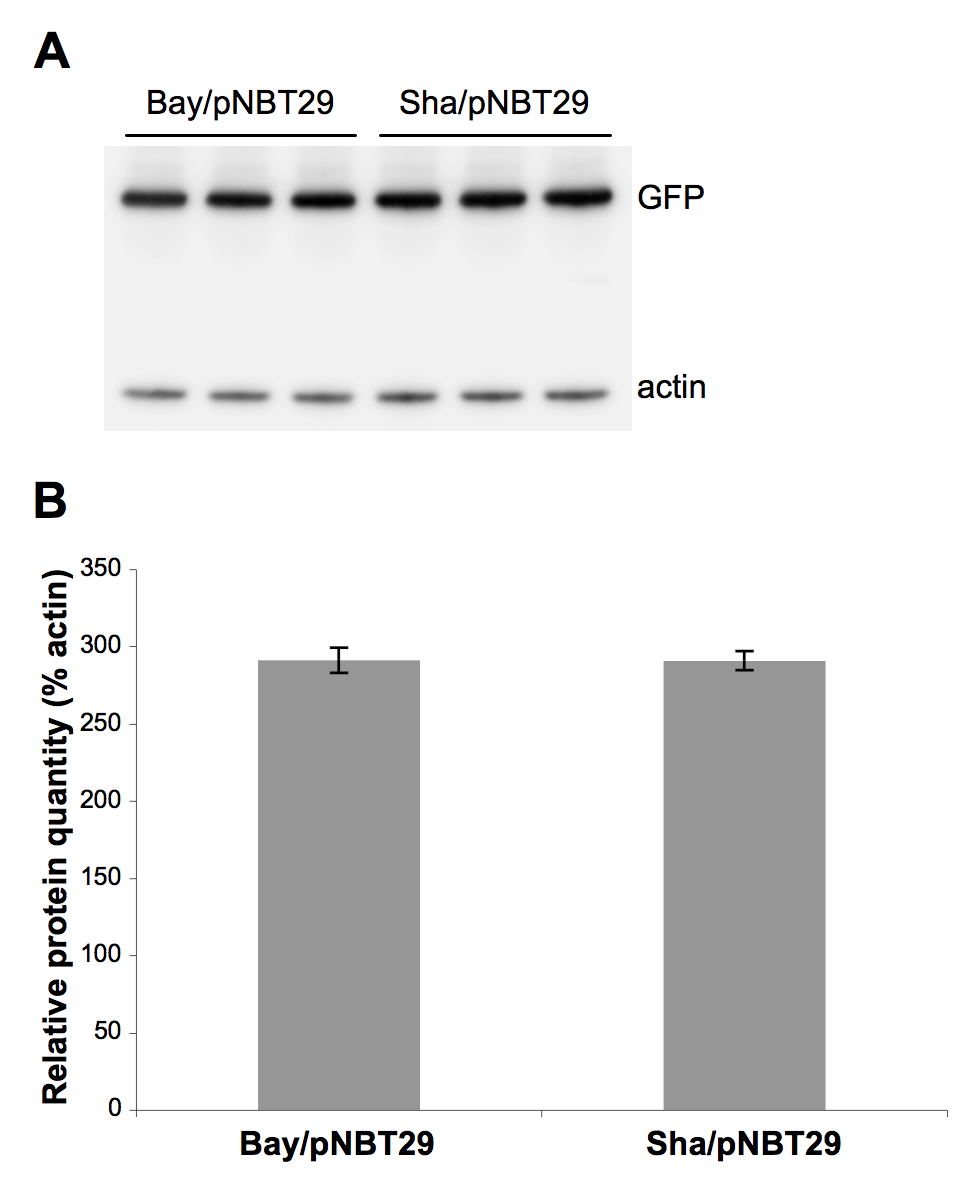

Supplement: Figure S4 — Yeast cells transformed with Bay/pNBT29 or Sha/pNBT29 express a comparable level of the two KCS18 isoforms. A, Western blot on protein extracts from yeast cells expressing Bay/pNBT29 or Sha/pNBT29 was performed using antibodies raised against GFP and actin as control. Three replicates were probed for each protein extracts. B, Comparison of the relative KCS18 protein quantity measured from the luminescence of the peroxidase activity. (TIF) [file pone.0049261.s004.tif]

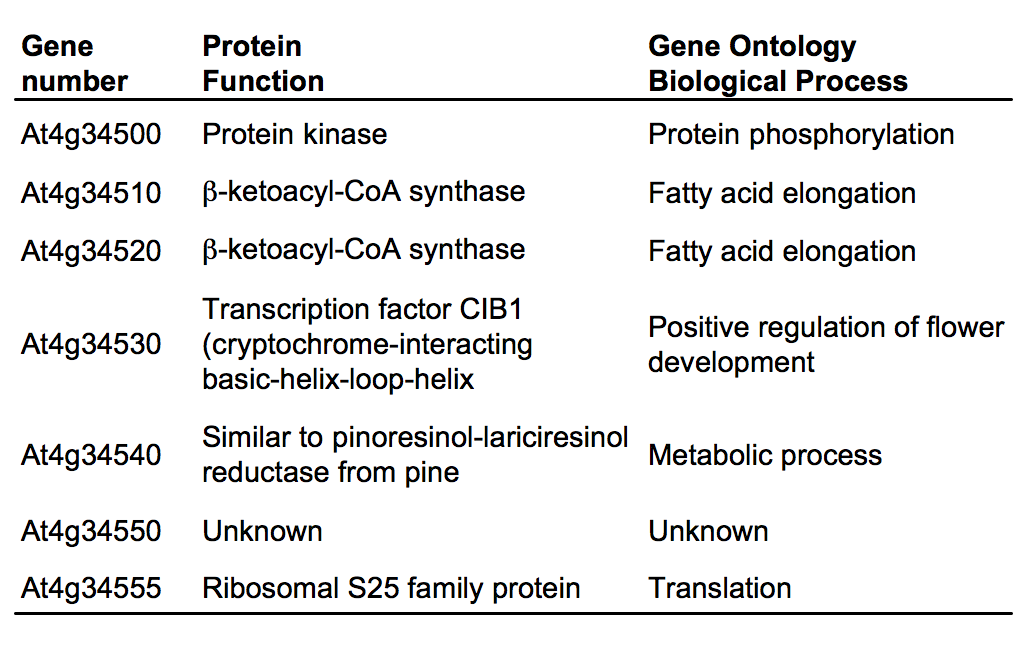

Supplement: Table S1 — List of genes in the CLR.2 18-kb candidate interval. (TIF) [file pone.0049261.s005.tif]

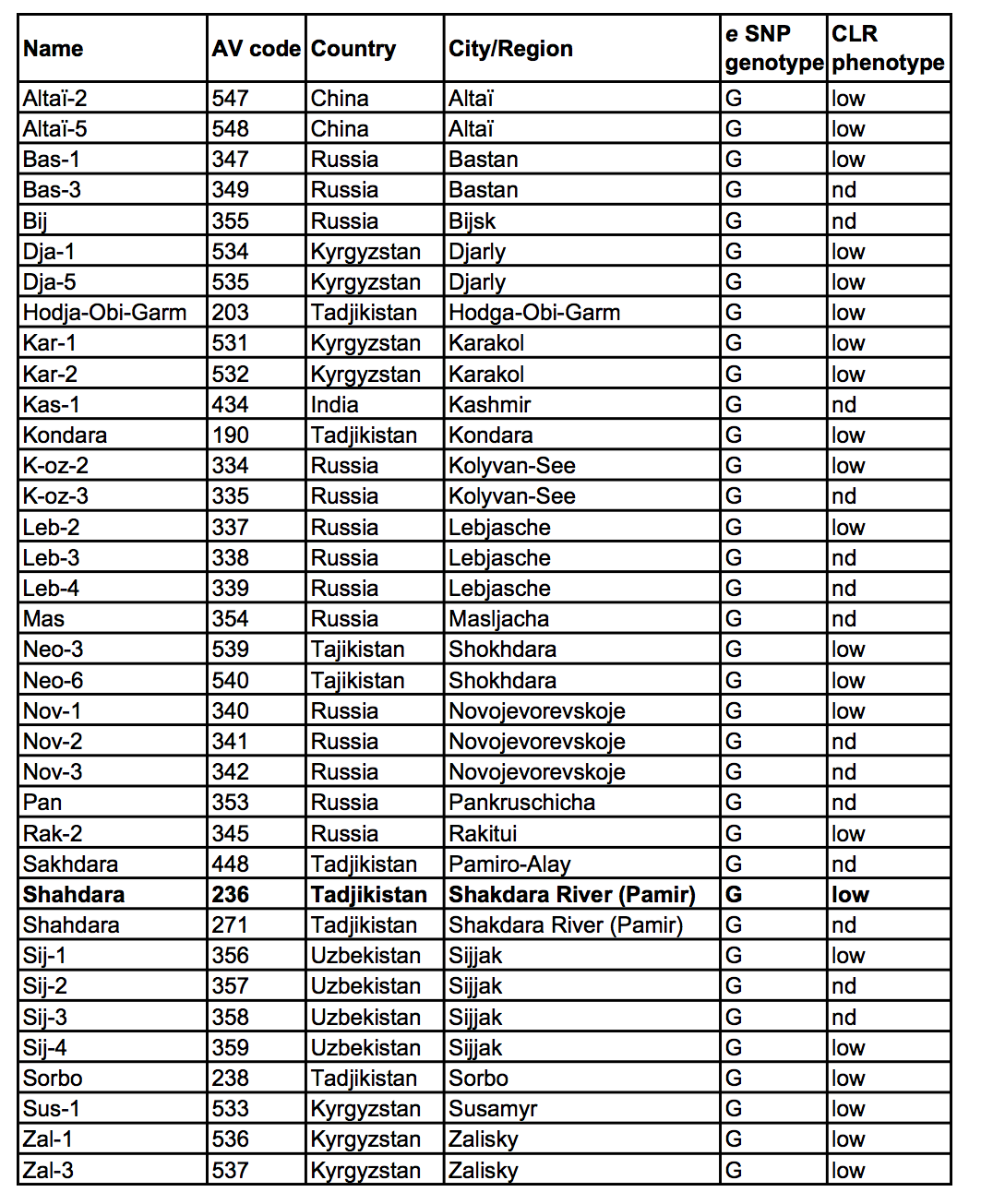

Supplement: Table S2 — List of Sha-like accessions. The name and the AV code of the accessions are mentioned with the country and city/region where they have been collected. nd, not determined. A low CLR phenotype means ≤0.33. (TIF) [file pone.0049261.s006.tif]

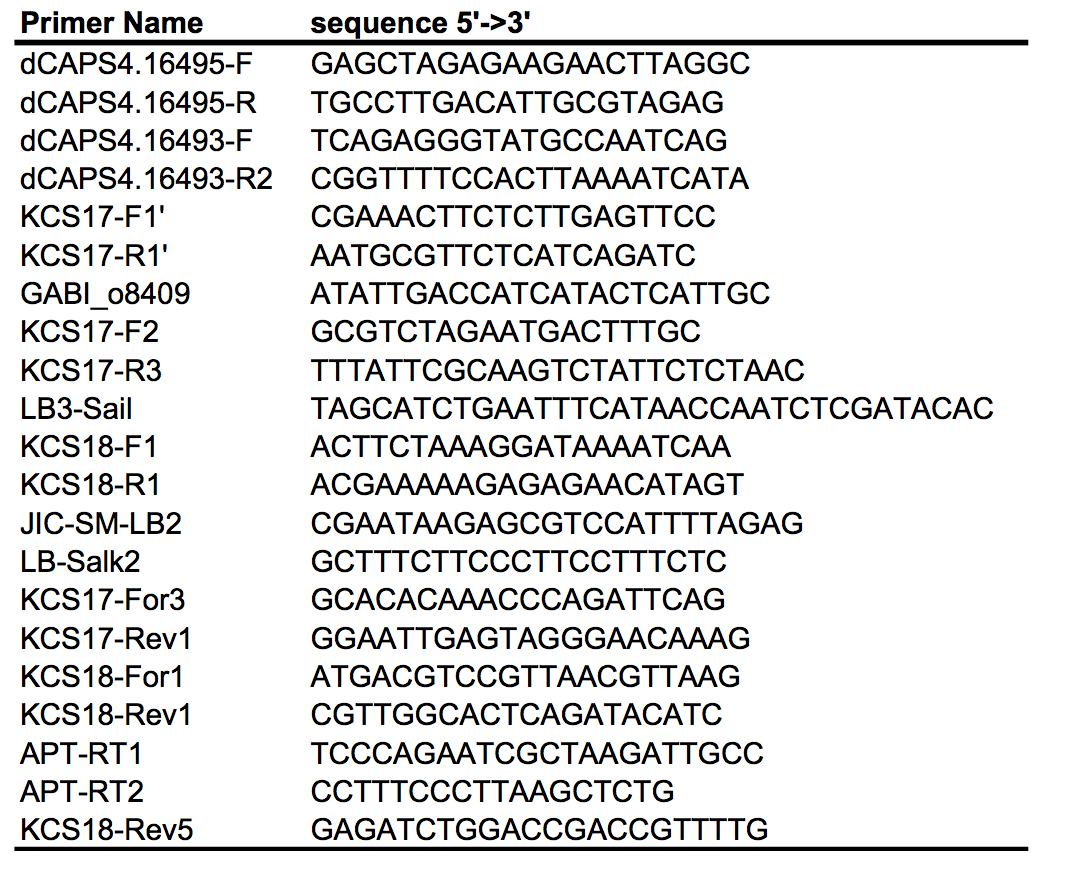

Supplement: Table S3 — List of primers used. (TIF) [file pone.0049261.s007.tif]
